# Long noncoding RNA TINCR facilitates hepatocellular carcinoma progression and dampens chemosensitivity to oxaliplatin by regulating the miR-195-3p/ST6GAL1/NF-κB pathway

**DOI:** 10.1186/s13046-021-02197-x

**Published:** 2022-01-03

**Authors:** Jie Mei, Wenping Lin, Shaohua Li, Yuhao Tang, Zhiwei Ye, Lianghe Lu, Yuhua Wen, Anna Kan, Jingwen Zou, Chengyou Yu, Wei Wei, Rongping Guo

**Affiliations:** 1 Department of Liver Surgery, Sun Yat-sen University Cancer Center, Guangzhou, China; 2 State Key Laboratory of Oncology in South China, Collaborative Innovation Center for Cancer Medicine, Guangzhou, China; 3 Department of Oncology, The First Affiliated Hospital of Sun Yat-sen University, Guangzhou, 510080 China

**Keywords:** TINCR, MiR-195-3p, ST6GAL1, Hepatocellular carcinoma, Oxaliplatin

## Abstract

**Background:**

Long non-coding RNAs (lncRNA) have an essential role in progression and chemoresistance of hepatocellular carcinoma (HCC). In-depth study of specific regulatory mechanisms is of great value in providing potential therapeutic targets. The present study aimed to explore the regulatory functions and mechanisms of lncRNA TINCR in HCC progression and oxaliplatin response.

**Methods:**

The expression of TINCR in HCC tissues and cell lines was detected by quantitative reverse transcription PCR (qRT-PCR). Cell proliferation, migration, invasion, and chemosensitivity were evaluated by cell counting kit 8 (CCK8), colony formation, transwell, and apoptosis assays. Luciferase reporter assays and RNA pulldown were used to identify the interaction between TINCR and ST6 beta-galactoside alpha-2,6-sialyltransferase 1 (ST6GAL1) via miR-195-3p. The corresponding functions were verified in the complementation test and in vivo animal experiment.

**Results:**

TINCR was upregulated in HCC and associated with poor patient prognosis. Silencing TINCR inhibited HCC proliferation, migration, invasion, and oxaliplatin resistance while overexpressing TINCR showed opposite above-mentioned functions. Mechanistically, TINCR acted as a competing endogenous (ceRNA) to sponge miR-195-3p, relieving its repression on ST6GAL1, and activated nuclear factor kappa B (NF-κB) signaling. The mouse xenograft experiment further verified that knockdown TINCR attenuated tumor progression and oxaliplatin resistance in vivo.

**Conclusions:**

Our finding indicated that there existed a TINCR/miR-195-3p/ST6GAL1/NF-κB signaling regulatory axis that regulated tumor progression and oxaliplatin resistance, which might be exploited for anticancer therapy in HCC.

**Supplementary Information:**

The online version contains supplementary material available at 10.1186/s13046-021-02197-x.

## Introduction

Hepatocellular carcinoma (HCC), the most common type of liver malignancies, is the third leading cause of cancer-related deaths worldwide [1]. Due to its concealed onset and rapid progression, HCC often develops to huge tumor burden, vascular invasion, and extrahepatic metastasis at time of diagnosis [2]. Traditional systemic platinum-based chemotherapy presents unsatisfactory efficacy and is not recommended in the treatment of late-staged HCC [3]. Recently, several studies confirmed the effectiveness of oxaliplatin-based chemotherapy for improving survival in unresectable HCC patients [4–7]. However, limited by the essentially strong ability of proliferation, metastasis, and drug resistance of HCC cells, some patients still present rapid progression after oxaliplatin therapy [8]. Thus, there is an urgent need to elucidate the mechanism of HCC progression and chemotherapy resistance, to find effective biomarkers to predict prognosis and improve treatment strategies.

Long non-coding RNAs (lncRNAs), defined as transcripts of more than 200 nucleotides that are not translated into proteins, have been associated with diverse functions [9]. To date, there has been an explosion of research focused on investigating the role of multiple lncRNAs in regulating proliferation, migration, expansion, and immortality of malignancies [10, 11]. Despite the elucidation of potential mechanistic roles, the biological relevance of the vast majority of lncRNAs remains uncertain [12]. Especially, the relationship between lncRNAs and cancer therapy resistance has received increased attention due to the complicated interaction network [13]. Take several well-known lncRNAs in HCC as examples, metastasis-associated lung adenocarcinoma transcript 1 (MALAT1) was investigated to promote tumor metastasis and resistance to 5-FU, DOX, and mitomycin [14]. Moreover, highly upregulated lncRNA in HCC (HULC) suppresses tumor proliferation and invasion, and upregulates the chemosensitivity of oxaliplatin, 5-FU and pirarubicin by inducing autophagy pathway [15]. This compelling evidence inspired us to explore the mechanisms of lncRNAs on regulating tumor progression and chemosensitivity in HCC.

Terminal differentiation-induced non-coding RNA (TINCR) is one of the most highly induced lncRNAs during epidermal differentiation [16]. It has been reported to be involved in the progression of many cancers [17]. Recently, TINCR was indicated to strengthen cisplatin resistance of nasopharyngeal carcinoma [18]. However, in HCC, the cognition of biological regulation mechanism of TINCR is limited, and its influence on chemosensitivity to oxaliplatin remains unknown.

In this study, we identified that TINCR is upregulated in HCC tissues and associated with poor patient prognosis. Further functional experiments showed that TINCR promotes HCC cells proliferation, invasion, metastasis, and oxaliplatin resistance. As a competing endogenous RNA (ceRNA), TINCR sponges miR-195-3p to increase ST6GAL1 expression and activate the nuclear factor kappa B (NF-κB) pathway. Our study elucidates that TINCR act as a promising biomarker and the regulatory mechanism provides potential therapeutic targets for HCC patients.

## Materials and methods

### Clinical specimens

The tumors and normal liver tissues were obtained from HCC patients who received hepatectomy in Sun Yat-sen University Cancer Center (SYSUCC). Detailed clinicopathological data were collected according to patient medical records. The long-term follow-up interval was from June 2010 to December 2020. This study was conducted according to the ethical guidelines of the 1975 Declaration of Helsinki, and approved by the Institutional Ethical Review Board of the SYSUCC (B2019–057-01).

### Cell culture

The human HCC cell lines (HepG2, HuH7, MHCC-LM3, MHCC-97H, MHCC-97 L, PLC/PRF/5, SK-HEP-1, Hep3B, SMMC-7721, SNU-449) were cultured in DMEM (Invitrogen) supplemented with 10% fetal bovine serum (Gibco) in an incubator sustaining an atmosphere of 5% CO2 and 37 °C. All the cell lines were purchased from the Cell Lines Service (Cellcook Biotech Co., Ltd., Guangzhou, China).

### RNA extraction, reverse transcription, and quantitative reverse transcription PCR

Total RNA was purified using Trizol (Invitrogen) or RNA quick purification kit (ESscience, China). Reverse transcription was performed using miRNA 1st Strand cDNA Synthesis Kit (Vazyme) with Bulge-Loop miRNA-specific RT primers (RiboBio) for miRNA or random primers (Promega) for mRNA and lncRNA. The primers used in qRT-PCR are listed in Supplementary Table S1. The relative expression level was compared with that of β-actin and fold changes were calculated using the 2^-△△ct^ method.

### Transient transfection and stable transfection of cell lines

Lipofectamine 3000, RNAiMAX, or Opti-MEM I reagents (Invitrogen) were used for transient transfection. The siRNA targeting TINCR, ST6GAL1 were synthesized and purchased from GenePharma. The sequence of TINCR, ST6GAL1, and nonsensical fragment were synthesized and cloned into pcDNA3.1 by Umine Biotechnology Co., LTD (Guangzhou). The mimics and inhibitors of miR-195-3p were designed and supplied by RiboBio. Stably transfected cell lines were constructed and purchased from Obio Technology Corp. All the sequence is listed in Table S2. Conventionally, transfection systems were co-cultured with targeted cells for 12 h, and then changed to normal medium.

### RNA sequencing and bioinformatic analysis

Total RNA of HepG2 cells transfected with anti-TINCR siRNAs was extracted to perform RNA sequencing by PGEM Biotechnology Co., Ltd. (Guangzhou). Differentially expressed genes and microRNAs were identified, and were conducted with Kyoto Encyclopedia of Genes and Genomes (KEGG) pathway analysis using the DAVID software. Gene set enrichment analysis (GSEA) was performed through GEO Common Dataset to identify biological functions enriched in HCC with TINCR knockdown. A threshold of *P* < 0.05 and an FDR ≤ 0.25 were used to select significant items.

### Cell proliferation and oxaliplatin sensitivity assay

Transfected cells (2 × 10^3^) were seeded in 96-well plates, and cell viability was detected every 24 h for 5 days using cell counting kit-8 (cck-8) (Glpbio). In the colony formation experiment, transfected cells (4 × 10^2^) were seeded in 6-well plates with and incubated for 14 days, and then fixed, stained, and counted. In addition, the cells were incubated with oxaliplatin (0, 0.5, 1, 2, 4, 8, 16, and 32 μg/ml) for 72 h and then tested for cell viability. In apoptosis assay, the cells were incubated with oxaliplatin at the right concentration for 24 h and then collected for flow cytometry assay.

### Flow cytometry assay

Transfected cells were digested with 0.25% trypsin and washed twice with PBS, adjusting to a concentration of 1 × 10.^6^ Next, 10 μl of Annexin V-FITC and 10 μl propidium staining solution (ESscience, China) were mixed and added to a 0.1 ml cell suspension and incubated at room temperature for 5 min in the dark. Apoptosis was detected by flow cytometry (FACSVantageSE, BD, USA).

### In vitro migration and invasion assays

Transwell chambers (Corning) coated without or with matrigel (Corning) were prepared for cell migration and invasion, respectively. Cells (1 × 10^5^) suspended in 200 μl of serum-free medium were seeded in the upper chamber, while 600 μl medium mixed with 10% FBS was added to the lower chamber. After 24 h, the cells that migrated/invaded to the lower surface of the membrane were observed and counted.

### Western blotting

Cells were collected and lysed in ice-cold RIPA lysis buffer (Sigma) containing protease and phosphatase inhibitor (Selleck) for 15 min. The supernatant was collected after centrifugation at 14000×g for 15 min. The protein concentration was determined using a BCA protein quantification kit (Beyotime). Then, 30 μg of total protein was added to a 10% SDS-PAGE gel and transferred to a 0.25-μm PVDF membrane (Bio-Rad). The PVDF membrane was blocked with 5% skim milk for 1 h at 37 °C. Next, the membrane was incubated with primary antibodies against GAPDH (Abcam, ab8245), ST6GAL1 (Proteintech, 14,355–1-AP), IκBα (Cell Signaling Technology, no.4814), p-IκBα (Cell Signaling Technology, no.2859), p65(Cell Signaling Technology, no.3031), p-p65(Cell Signaling Technology, no.3033) overnight at 4 °C. After incubation with horseradish peroxidase (HRP)-labeled secondary antibodies at 37 °C for 30 min, the bands were detected using enhanced chemiluminescence (GBCBIO, China) with the ChemiDoc MP Imaging System (Bio-Rad).

### Luciferase reporter assays

The wild-type or mutant fragment of TINCR or ST6GAL1 containing the predicting binding sequence of miR-195-3p was subcloned into a psiCHECK2 Dual-luciferase vector (Promega). To verify the target genes of miRNAs, HCC cells were co-transfected with luciferase reporter plasmids and miR-195-3p mimics or negative control. Luciferase activity was measured using the dual-luciferase reporter assay system (Promega). Renilla luciferase expressed by pRL-PGK (Promega) was used as an internal control to correct for differences in both transfection and harvest efficiency.

### RNA pulldown

The biotin-labeled miRNA pull-down assay was performed using the RNA Pulldown Kit (BersinBio, Guangzhou, China). Briefly, cells were transfected with biotin-labeled miR-195-3p and miR-ctrl (RiboBio, 50 nM), and lysed after 48 h incubation. Simultaneously, 25 μg streptavidin magnetic beads were mixed with sample lysates and incubated with rotation overnight at 4 °C. Beads were then washed to remove unbound materials. RNA was eluted, isolated, and subjected to qRT-PCR analysis.

### Mouse xenograft models

BALB/c nude mice (4–5 weeks old, female) were provided by Guangdong Animal Experiment Center. For tumor growth model, sh-TINCR and scrambled control cells (4 × 10^6^) were resuspended in 100 μl PBS containing 10% matrigel (Corning), and then subcutaneously injected into either side of the posterior flank of the same mouse. When tumor volume reached approximately 100 mm^3^, the mice were randomly divided into four groups (*n* = 7), and intraperitoneally injected with normal 5% glucose solution or oxaliplatin (Selleck, 3 mg/kg) every 3 days. Tumors were measured every 3 days from the start of dosing. On day 24, mice were sacrificed and tumors were detached for further measurement and immunohistochemistry detection. All animal experiments complied with the 1978 National Institutes of Health guide for the care and use of laboratory animals. The animal procedures were approved by the Institutional Animal Care and Use Committee of SYSUCC (L102042019110H).

### Immunohistochemical (IHC)

Paraffin-embedded tumor tissues were cut as 5-μm sections and processed for IHC. Tissue sections were prepared for antigen retrieval using microwave treatment in citrate buffer (pH 8.0, Beyotime) and then incubated with anti-ST6GAL1 antibody (Proteintech, 14355–1-AP) overnight at 4 °C. Immunostaining was performed using the Envision Inspection System with diaminobenzidine as substrate (DAKO Cytomation, Glostrup, Denmark).

### Statistical analysis

Categorical variables were compared using chi-squared test or Fisher exact test. Difference between groups was determined by the Student’s test for one single comparison, and by the ANOVA test for multiple comparisons. Difference between groups was determined using the Student’s t-test. Survival analysis was performed using the Kaplan-Meier method, and differences in the survival curves were analyzed with the log-rank test. Univariate and multivariate Cox regression analyses were performed to determine prognostic factors for overall survival (OS), and recurrence-free survival (RFS). Hazard ratios (HRs) and confidence intervals (CI) were also calculated. Spearman correlation analysis was used to calculate the correlation between TINCR and ST6GAL1 expression. A two-tailed *P*-value < 0.05 was considered statistically significant. All data analyses were performed using SPSS 25.0 software (SPSS Inc., Chicago, IL), GraphPad Prism (version 8.0, GraphPad Software, Inc.), and R version 4.0.2.

## Result

### TINCR is upregulated in HCC and associated with poor patient prognosis

**Fig. 1:**
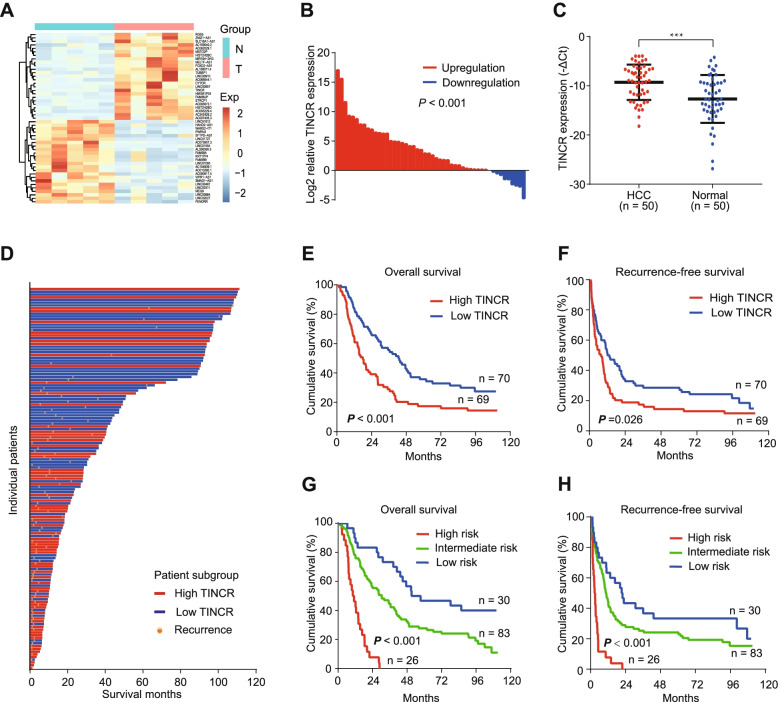
TINCR is upregulated in HCC and correlates with poor patient prognosis. **A**, Heat map of the most differentially expressed lncRNAs between five pairs of HCC and normal liver tissues (fold change > 2; *P* < 0.01). **B**, Expression of TINCR in HCC compared to that in normal liver tissues (50 pairs). **C**, Expression of TINCR in HCC (*n* = 50) and normal liver tissues (n = 50). Data represent the mean ± SEM. **D**, Waterfall plot of patients according to high or low TINCR expression. E-F, Kaplan–Meier analysis of overall (**E**) and recurrence-free (I) survival in high TINCR (*n* = 70) and low TINCR (*n* = 69) groups. **G**-**H**, Risk stratification of overall (**G**) and recurrence-free (**H**) survival based on the prognostic model integrating albumin-bilirubin grade, tumor size, macrovascular invasion, and TINCR expression. Low risk, score 0, *n* = 30; intermediate risk, score 1–2, *n* = 83; and high risk, score 4–5, *n* = 26. For (**B**) to (**H**), TINCR expression was measured using quantitative qRT-PCR. GAPDH was used as an internal control. ***P < 0.001

To identify lncRNAs that regulate HCC progression, we performed a full transcriptome sequence on 5 pairs of tumor and normal tissues from 5 HCC patients. 49 lncRNAs with different expressions were found (fold change > 2, FDR < 0.05, and *P* < 0.01; Fig. 1A), of which TINCR was one of the significant lncRNAs upregulated in HCC. In addition, we reconfirmed that TINCR expression was significantly higher in 50 HCC tissues compared to 50 normal tissues by qRT-PCR (Fig. 1B-C; Fig. S1A, *P* < 0.001).

We further investigated the prognostic value of TINCR with 139 HCC frozen HCC tissues using qRT-PCR. According to the median value of TINCR expression, the patients were divided into high and low expression groups. The result showed that patients in the high TINCR group were associated with worse overall survival (Fig. 1D-E, *P* < 0.001) and recurrence-free survival (Fig. 1F, *P* < 0.026). In addition, combined with the clinical data of the patients, we surprisingly found that TINCR expression was not related to those generally recognized risk factors, including tumor size, number, vascular invasion, degree of differentiation (Table. S3). However, the univariate and multivariate analysis suggested that TINCR expression was an independent prognostic factor, which further confirmed the unique biological regulatory function of TINCR (Table. S4–5).

We then constructed a prognostic model integrating albumin-bilirubin grade, tumor size, macrovascular invasion, and TINCR expression, and HCC patients were divided into low, intermediate, and high-risk groups (Table. S6). Survival plots showed significant stratification in the overall and recurrence-free survival among the three groups (Fig. 1G-H, both *P* < 0.001).

### TINCR promotes HCC cell proliferation, migration, invasion, and resistance to oxaliplatin

**Fig. 2:**
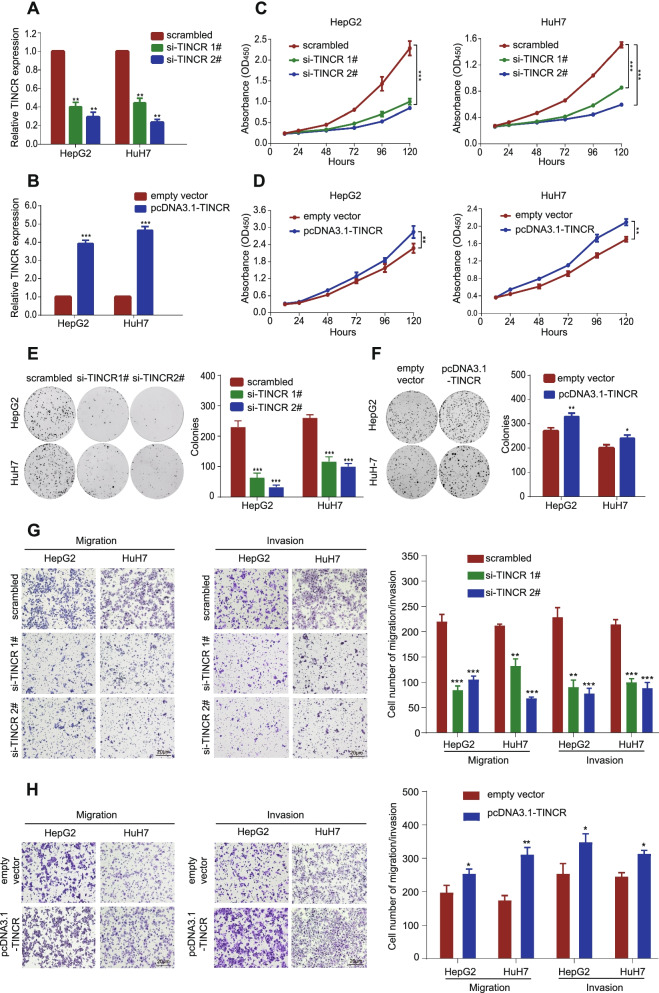
TINCR promotes HCC cell proliferation, migration, and invasion, and resistance to oxaliplatin. **A**-**B**, TINCR expression in HepG2 and HuH7 cells transfected with si-TINCRs or scrambled control (**A**), and pcDNA3.1-TINCR or empty vector (**B**). **C**-**D**, CCK-8 assay of HepG2 and HuH7 cells transfected with si-TINCRs or scrambled control (**C**), and pcDNA3.1-TINCR or empty vector (**D**). **E**-**F**, Representative images (left) and quantification (right) of the colony formation assay in HepG2 and HuH7 cells transfected with si-TINCRs or scrambled control (**E**), and pcDNA3.1-TINCR or empty vector (**F**). **G**-**H**, Representative images (left) and quantification (right) of transwell migration and invasion assays in HepG2 and HuH7 cells transfected with si-TINCRs or scrambled control (**G**), and pcDNA3.1-TINCR or empty vector (**H**). Data are expressed as the mean ± SD of at least three independently repeated experiments. *P < 0.05; **P < 0.01; ***P < 0.001

**Fig. 3:**
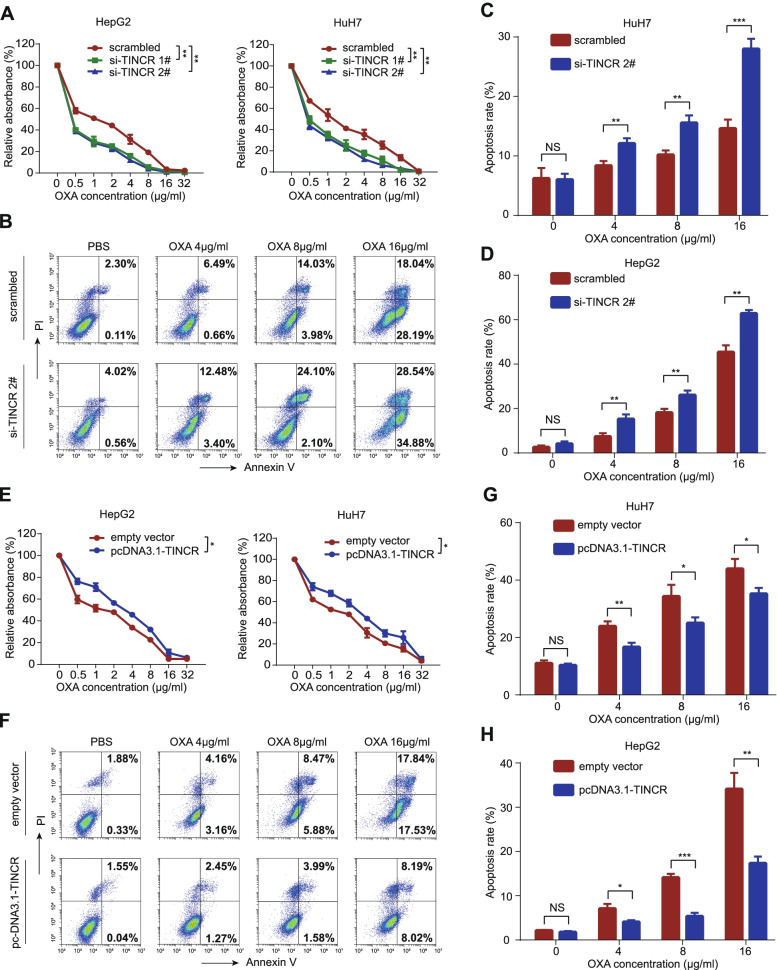
TINCR enhances the resistance to oxaliplatin in HCC cells. **A**, The survival percentage of HepG2 and HuH7 cells transfected with si-TINCRs or scrambled control, treated with increasing concentration of oxaliplatin (OXA). **B**-**D**, Representative images of apoptosis assay of increasing concentration in HepG2 cells transfected with si-TINCRs or scrambled control (**B**), and quantification in HuH7 (**C**) and HepG2 (**D**) cells. **E**, The survival percentage of HepG2 and HuH7cells transfected with pcDNA3.1-TINCR or empty vector, treated with increasing concentration of OXA. **F**-**H**, Representative images apoptosis assays of increasing concentration of OXA in HepG2 cells transfected with pcDNA3.1-TINCR or empty vector (**F**), and quantification in HuH7 (**G**) and HepG2 (**H**) cells. Data are expressed as the mean ± SD of at least three independently repeated experiments. *P < 0.05; **P < 0.01; ***P < 0.001; NS: no significance

The expression of TINCR was evaluated in various common HCC cell lines using qRT-PCR (Fig. S1B). Given the generally low expression value of TINCR in HCC cell lines, HuH7 and HepG2, with relatively higher expression of TINCR compared with other cell lines, were selected as candidates for further functional exploration. We transfected HuH7 and HepG2 cells with two siRNAs against TINCR (si-TINCR 1# and si-TINCR 2#) and TINCR-overexpressing plasmid (pcDNA3.1-TINCR). The transfection efficiency was detected using qRT-PCR (Fig. 2A-B). CCK-8 and colony formation assays showed significant inhibition of proliferation of HCC cells transfected with si-TINCR compared to those transfected with scrambled control (Fig. 2C and E, all *P* < 0.001), similarly the opposite effects were observed in HCC cells transfected with pcDNA3.1-TINCR compared to those transfected with empty vector (Fig. 2D and F, *P* < 0.05). In addition, silencing TINCR inhibited HCC cells migration and invasion (Fig. 2G, all *P* < 0.01), while overexpressing TINCR enhanced those cell behaviours (Fig. 2H, all *P* < 0.05). Moreover, we examined the role of TINCR in the regulation of oxaliplatin sensitivity. Under different concentration gradients of oxaliplatin, CCK-8 and apoptosis assays revealed that silencing TINCR could significantly enhance the sensitivity of HCC cells to oxaliplatin (Fig. 3A-D; Fig. S1C, all *P* < 0.01). Furthermore, the opposite effect was verified in HCC cells with overexpression (Fig. 3E-H; Fig. S1D, all *P* < 0.05). These findings indicated that TINCR acts as an oncogenic lncRNA to promote proliferation, invasion, and migration, and induces sensitivity to oxaliplatin in HCC.

### TINCR acts as a ceRNA and competitively absorbs miR-195-3p

**Fig. 4:**
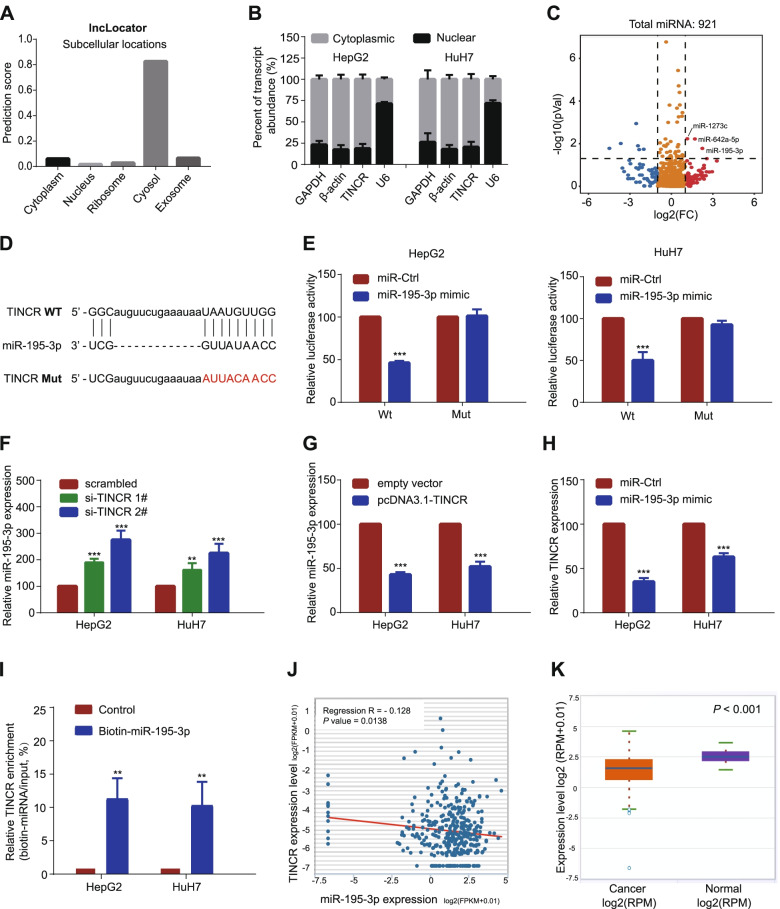
TINCR acts as a ceRNA and competitively absorbs miR-195-3p. **A**, TINCR was predicted to be located mainly in the cytosol using the bioinformatics tools in LncLocator. **B**, quantitative reverse transcription PCR analysis of TINCR expression in the nucleus and cytoplasm of HepG2 and HuH7 cells. GAPDH, β-actin, and U6 were used as endogenous controls. **C**, Volcano plots selected the significant up-regulated miRNAs in TINCR-silencing HCC cells (fold change > 1.5; P < 0.05). **D**, The predicted miR-195-3p binding sites in the TINCR transcript. The red nucleotides represent mutant sequences of target sites. **E**, The luciferase activities in HepG2 and HuH7 cells. **F**-**G**. Relative levels of miR-195-3p in HepG2 and HuH7 cells transfected with si-TINCRs or scrambled control (**F**), and pcDNA3.1-TINCR or empty vector (**G**). **H**, Relative levels of TINCR in HepG2 and HuH7 cells transfected with miR-195-3p mimic or miRNA control. **I**, Enrichment of TINCR pulled down by biotin-miR-195-3p or negative control. **J**, The relationship between levels of TINCR and miR-195-3p in HCC in starBase. **K**, MiR-195-3p was down-regulated in HCC compared to that in normal liver issues. Data are expressed as the mean ± SD of at least three independently repeated experiments. *P < 0.05; **P < 0.01; ***P < 0.001

To figure out the underlying regulatory mechanism, we firstly explored the predictive location of TINCR in LncLocator (http://www.csbio.sjtu.edu.cn/bioinf/lncLocator). TINCR was presumed to be located mainly in the cytoplasm (Fig. 4A). QRT-PCR analysis of TINCR expression in the nucleus and cytoplasm further confirmed this conclusion (Fig. 4B). Based on these results, we hypothesized that TINCR functions as a ceRNA. To find the binding miRNAs, we performed the sequencing of the miRNA of HepG2 with TINCR knockdown compared with control treatment and found the miR-195-3p was one of the significantly increased miRNAs with the most maximum fold change value (Fig. 4C). Next, dual-luciferase reporter assays showed that overexpression of miR-195-3p reduced luciferase activity of the wild-type TINCR gene fragment, however, not the mutant TINCR vector (Fig. 4D-E, *P* < 0.001). We further verified the opposite expression value of TINCR and miR-195-3p through qRT-PCR in HCC cells. The results showed that miR-195-3p was upregulated with suppression of TINCR, while downregulated with overexpression of TINCR (Fig. 4F-G, all *P* < 0.001). In contrast, TINCR was downregulated when miR-195-3p was overexpressed (Fig. 4H, *P* < 0.001). RNA pulldown assay showed a conspicuous elevated ratio of TINCR expression to total input in HCC cells transfected with biotin-labeled miR-195-3p compared with that in the control (Fig. 4I, P < 0.01). Finally, findings from the TCGA database and Starbase (http://starbase.sysu.edu.cn/index.php) indicated that miR-195-3p was negatively correlated with TINCR, and might serve as a tumor suppressor molecule with an opposite trend in clinicopathological characteristics and survival prediction to TINCR (Fig. 4J-K; Fig. S2A). The above results suggested that the carcinogenic effect of TINCR is partially mediated by the negative regulation of miR-195-3p.

### TINCR relieves the repression of miR-195-3p on ST6GAL1, enhancing the NF kappa B pathway

**Fig. 5:**
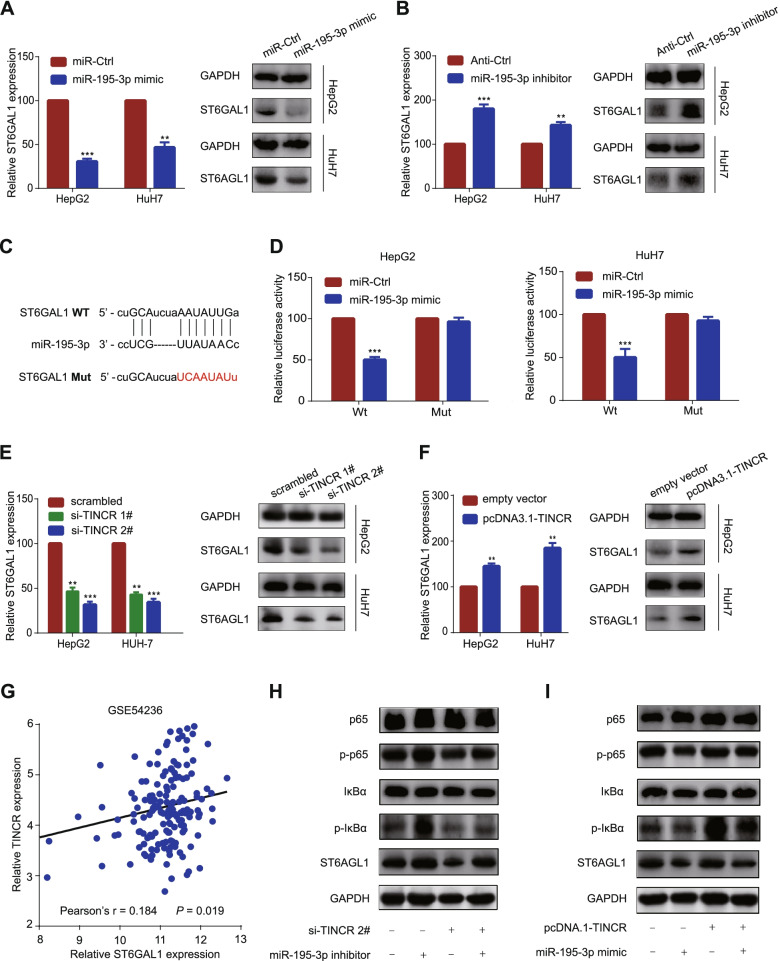
TINCR relieves the repression of miR-195-3p on ST6GAL1, enhancing the NF kappa B Pathway. **A**, Relative mRNA (left) and protein (right) levels of ST6GAL1 in HepG2 and HuH7 cells transfected with miR-195-3p mimic or miR-Ctrl. **B**, Relative mRNA (left) and protein (right) levels of ST6GAL1 in HepG2 and HuH7 cells transfected with miR-195-3p inhibitor or anti-Ctrl. **C**, The predicted miR-195-3p binding sites in the ST6GAL1 transcript. The red nucleotides represent mutant sequences of target sites. **D**, The luciferase activities in HepG2 and HuH7 cells. **E**, Relative mRNA (left) and protein (right) levels of ST6GAL1 in HepG2 and HuH7 cells transfected with si-TINCRs or scrambled control. **F**, Relative mRNA (left) and protein (right) levels of ST6GAL1 in HepG2 and HuH7 cells transfected with pcDNA3.1-TINCR or empty vector. **G**, Correlation between TINCR and ST6GAL1 expression based on the GEO database (GSE54236). **H**-**I**, Western blot analysis of expression of ST6GAL1 and phosphorylation levels of IκBα (p-IκBα) and p65 (p-p65) in HepG2 cells cotransfected with si-TINCR #2 or scrambled control together with miR-195-3p inhibitor or anti-Ctrl (**H**), and pcDNA3.1-TINCR or empty vector together with miR-195-3p mimic or miR-Ctrl (**I**). Data are expressed as the mean ± SD of at least three independently repeated experiments. *P < 0.05; **P < 0.01; ***P < 0.001

To find the downstream target protein of miR-195-3p, we screened the target genes with a cumulative weighted context score less than − 0.1 in TargetScan (http://www.targetscan.org/). Among these predicted target genes, ST6GAL1 was the probable candidate gene, since it exhibited an upward trend according to the sequencing of mRNA of HepG2 with TINCR knockdown compared with control treatment (Fig. S2G), and was reported to be closely related to tumor progression and drug resistance in several studies [19]. We found that ST6GAL1 expression was affected by the change of miR-195-3p (Fig. 5A-B, *P* < 0.01). To validate this potential interaction, dual-luciferase reporter assays showed that overexpression of miR-195-3p reduced luciferase activity of the wild-type ST6GAL1 gene fragment, but not the mutant ST6GAL1 vector (Fig. 5C-D, *p* < 0.001). These results indicated that ST6GAL1 was the target gene of miR-195-3p.

In addition, western blotting revealed that the expression of ST6GAL1 was positively correlated with the expression of TINCR (Fig. 5E-F, *P* < 0.01), which was further validated using the GEO data set (GSE54236, Fig. 5G). To find the potential signaling pathway, GSEA analysis was performed based on the TCGA data set. The result showed significant enrichment of high TINCR expression on gene sets related to several classical pathways, including NF-κB pathway (Fig. S2B-C), while some typical related pathways were excluded after exploration (Fig. S2H). To verify this, results of further experiments revealed that ST6GAL1 was decreased after silencing TINCRs protein levels and the phosphorylation levels of IκBα (p- IκBα) and p65 (p-p65). The effects could be partially reversed by inhibition of miR-195-3p (Fig. 5H). In contrast, TINCR overexpression increased ST6GAL1 protein levels and the phosphorylation levels of IκBα (p- IκBα) and p65 (p-p65), which were partially reversed by overexpression of miR-195-3p (Fig. 5I). Based on these findings, we proposed that TINCR absorbed miR-195-3p to upregulate ST6GAL1, and activate the NF-κB pathway.

### ST6GAL1 is responsible for TINCR-mediated cell progression and oxaliplatin insensitivity

**Fig. 6:**
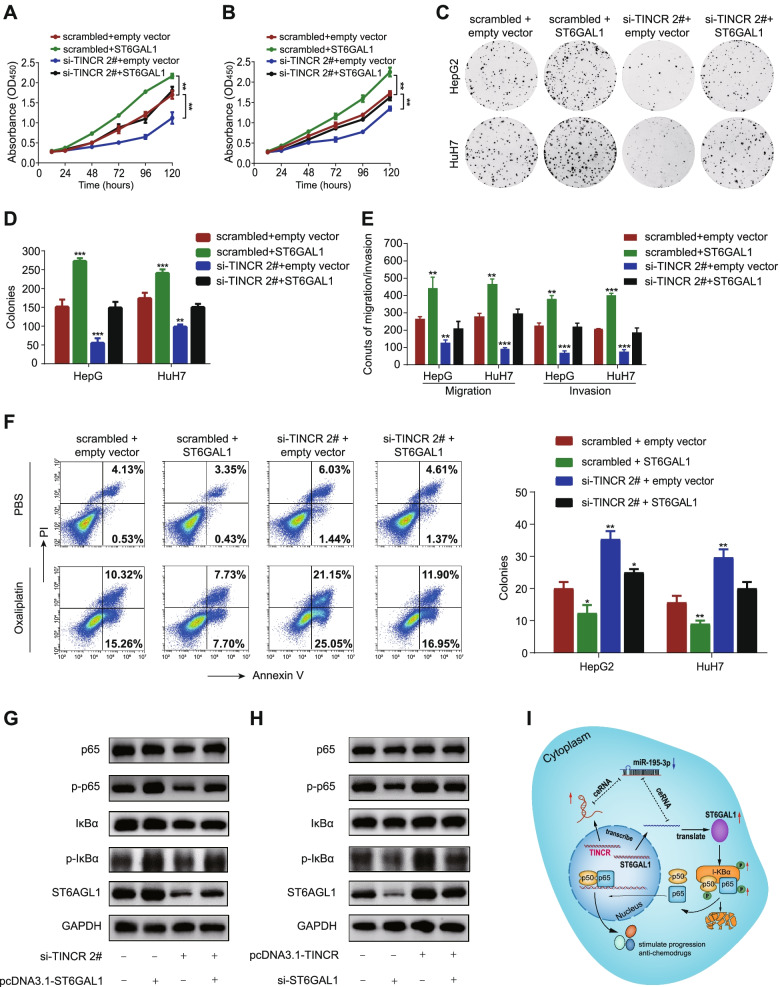
ST6GAL1 is responsible for TINCR-mediated cell progression and oxaliplatin insensitivity. **A**-**B**, CCK-8 assay of HepG2 (**A**) and HuH7 (**B**) cells cotransfected with si-TINCR 2# or scrambled control together with pc-DNA3.1-ST6GAL1 or empty vector. **C**-**D**, Representative images (**C**), and quantification (**D**) of the colony formation assay in the above-mentioned transfected cells. **E**, Quantification of transwell migration and invasion assays in the above-mentioned transfected cells. **F**, Representative images (left, only showed HepG2) and quantification (right) of the apoptosis assay of oxaliplatin (16 μg/ml) in transfected HepG2 and HuH7 cells. **G**-**H**, Western blot analysis of expression of ST6GAL1 and NF-kappa-B-related markers in HepG2 cells cotransfected with si-TINCR #2 or scrambled control together with pcDNA3.1-ST6GAL1 or empty vector (**G**), and pcDNA3.1-TINCR or empty vector together with si-ST6GAL1 or scrambled control (**H**). **I**, Mechanism model of the TINCR/miR-195-3p/ST6GAL1 axis and its effect on NF-kappa B Signaling. Data are expressed as the mean ± SD of at least three independently repeated experiments. *P < 0.05; **P < 0.01; ***P < 0.001

To confirm the TINCR-mediated function of ST6GAL1, we transfected HuH7 and HepG2 cells with ST6GAL1-overexpression vector or empty vector on basis of TINCR knockdown. Cell function assays revealed that restoration of ST6GAL1 expression partially rescued the reduced ability of proliferation, invasion, migration (Fig. 6A-E; Fig. S2D-E, *P* < 0.01), as well as oxaliplatin resistance (Fig. 6F; Fig. S2F, *P* < 0.05). Besides, restoration of ST6GAL1 also partially make up for the weakened phosphorylation levels of IκBα (p- IκBα) and p65 (p-p65), whereas overexpression of ST6GAL1 exhibited the opposite effect (Fig. 6G-H). In general, these finding further illustrated that TINCR promoted HCC cells progression and resistance to oxaliplatin via the TINCR/miR-195-3p/ST6GAL1/ NF-κB signaling pathway (Fig. 6I).

### Silencing TINCR impairs HCC tumorigenesis and resistance to oxaliplatin in vivo

**Fig. 7:**
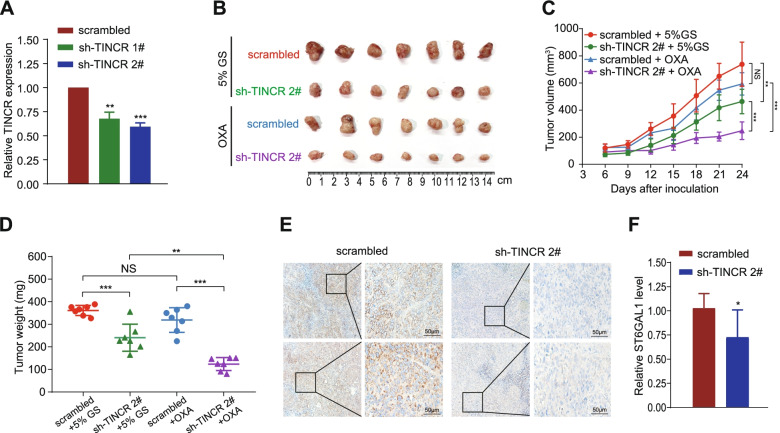
Silencing TINCR impairs HCC tumorigenesis and resistance to oxaliplatin in vivo. **A**, TINCR expression in HepG2 and HuH7 cells transfected with stable sh-TINCRs or scrambled control. **B**-**D**, HuH7 cells with or without stable TINCR knockdown were transplanted into the axilla of nude mice. Once the tumor nodes grew to 100 mm^3^, the mice were randomly divided into four groups (*n* = 6/group) and intraperitoneally injected with 5% glucose solution (GS) or oxaliplatin (OXA) (3 mg/kg) every 3 days. Representative images (**B**), tumor volume growth curves (**C**), and weight of the xenograft tumors(D). **E**-**F**, Representative images (**E**) and quantification (**F**) of immunohistochemistry analysis of ST6GAL1 expression level in tumors from HuH7 cells stably transfected with sh-TINCR 2# and scramble control. Data are expressed as the mean ± SD. *P < 0.05; **P < 0.01; ***P < 0.001; NS: no significance

To verify the oncogenic function of TINCR in vivo, nude mice were subcutaneously inoculated with TINCR silencing and control stable-transfected HuH7 cells to build xenograft mouse models. The degree of TINCR knockdown was confirmed by qRT-PCR (Fig. 7A). The growth of tumor size and weight in sh-TINCR group were strongly suppressed compared with that in scramble control group. Moreover, tumors in sh-TINCR group were significantly more sensitive to oxaliplatin treatment compared with that in ctrl-TINCR group (Fig. 7B-D, *P* < 0.01). Synchronously, ST6GAL1 expression in tumor tissues was reduced with the silencing of TINCR (Fig. 7E-F, *P* < 0.05). These data indicated that silencing TINCR could restrain HCC cell proliferation and oxaliplatin resistance in vivo.

## Discussion

Emerging evidence showed that lncRNAs played a crucial regulatory role in various types of carcinomas. In this study, we proposed that TINCR was upregulated in HCC tissues and indicated a gloomy prognosis in HCC patients. TINCR acted as a ceRNA to sponge miR-195-3p to facilitate ST6GAL1 expression, strengthening NF-κB signaling pathway, leading to promotion in HCC cell progression and oxaliplatin resistance in vitro and vivo. Our evidence suggests that TINCR may be served as a prognostic biomarker and potential therapeutic target for HCC patients.

The clinical significance of TINCR was previously reported in several malignant tumors, including HCC [17, 20]. However, our patient data showed that no correlation was found between TINCR expression and tumor size, tumor node metastasis, or vascular invasion. Excluding the possibility of individual particularity of the case, TINCR, independent of tumor stage-related factors, indicated the intrinsic malignant transformation of HCC cells, is of great significance. Besides, to our knowledge, for the first time, TINCR was found to be associated with oxaliplatin sensitivity in HCC. Accumulating evidence suggests that TINCR may be a promising prognostic biomarker to guide the development of personalized chemotherapies for HCC patients.

Accumulating evidence supports the existence of a typical interaction network involving ceRNAs [21], that is, lncRNAs regulate miRNAs by competitively binding to their target sites on protein-coding mRNA molecules. Given that the localization of TINCR in HCC is in the cytoplasm, we confirmed the regulatory axis of TINCR/miR-195-3p/ST6GAL1. The carcinogenic role of miR-195-3p remains uncertain. It was reported as an oncogene in renal cell carcinoma [22], but mostly played an anti-tumor role in various tumors. For example, miR-195-3p inhibited cervical cancer cell proliferation by targeting BCDIN3D [23], and reversed CCL4-enhanced VEGF-C expression in Oral Squamous Cell Carcinoma [24]. In HCC, miR-195-3p might be a critical negative modulator of UBE2I, leading to the restraint of metastasis [25]. The inhibiting effect of miR-195-3p in HCC was consistent with our findings.

As a ceRNA, the function of lncRNAs depends on the miRNA target. In this study, we proved the ST6GAL1 was the downstream target molecule of TINCR. ST6GAL1 encodes a member of glycosyltransferase family 29, which is a type II membrane protein that catalyzes the transfer of sialic acid from CMP-sialic acid to galactose-containing substrates [26]. Aberrant glycosylation is a universal feature of cancer cells, and ST6GAL1 is reported to be associated with aggressive, invasive disease with chemoresistance in numerous cancers [19]. The signaling pathways activated by ST6GAL1 mostly focused on PI3K/Akt, HIF1α, and TGFβ signaling [27–29]. Strengthened NF-κB signaling by ST6GAL1 in HCC could be supplementary evidence for enhancing progression and chemoresistance in cancer cells. To verify the specific activation way of p65, we used phosphorylation inhibitor (BAY 11–7085) to inhibit the p-IκBα. The p65 phosphorylation failed to be rescued by upregulating ST6GAL1 when the p-IκBα was blocked. (Fig. S2I). However, whether NF-κB is the unique pathway mediated by TINCR to induce oxaliplatin resistance needs to be further elucidated, and the downstream regulatory molecules await deeper investigation. Moreover, recently, ST6GAL1 was proven to be a serum biomarker that identifies lenvatinib-susceptible FGF19-driven HCC. Therefore, whether TINCR is related to tyrosine kinase inhibitors treatment in HCC is worth exploring. Given the potential widespread impact of TINCR and its downstream molecules, an improved understanding of how TINCR mediated sialylation controls cancer cell biology may give new horizons to a range of HCC therapeutics.

## Conclusions

In summary, this study identifies TINCR as an oncogenic lncRNA in HCC, and elucidates its function in promoting HCC cell proliferation, migration, invasion, and oxaliplatin resistance by TINCR/miR-195-3p/ST6GAL1 regulatory axis. Our data may facilitate a deeper understanding of the mechanisms associated with tumor progression and contribute to develop more effective therapeutic targets and biomarkers for chemotherapy in HCC patients.

## Supplementary Information

**Additional file 1: Figure S1** TINCR functions as an oncogenic lncRNA in HCC. A, Expression of TINCR in HCC compared to that in normal liver tissues (50 pairs). Paired t-test, *P* < 0.001. B, TINCR expression level in the 10 typical HCC cell lines. C-D, Representative images of apoptosis assay of increasing concentration of oxaliplatin in HuH7 cells transfected with si-TINCRs or scrambled control (C), and pcDNA3.1-TINCR or empty vector (D).

**Additional file 2: Figure S2**. TINCR regulates HCC progression and oxaliplatin sensitivity through TINCR/miR-195-3p/ST6GAL1/NF kappa B Signaling. A, MiR-195-3p tends to be associated with good prognosis in the TCGA database. B-C, GSEA analysis based on TCGA data set shows a significant enrichment of high TINCR expression on gene sets related to several classical pathways (B), including NF kappa B pathway (C). D-E, Quantification of transwell migration (D) and invasion (E) assays in HepG2 and HuH7 cotransfected with si-TINCR 2# or scrambled control together with pc-DNA3.1-ST6GAL1 or empty vector. F, Representative images of the apoptosis assay of oxaliplatin (16 μg/ml) in the above-mentioned transfected HuH7 cells. G, 7 genes including ST6GAL1 are identified between the predicted target mRNAs in TargetScan and mRNAs that down-regulated with TINCR silencing in RNA-Seq. H, Western blot analysis of expression of key molecules in other typical pathways in HepG2 cells transfected with scrambled, si-ST6GAL1 or si-TINCR #2. I, Western blot analysis of expression of ST6GAL1 and NF-kappa-B-related markers in HepG2 cells cotransfected with pcDNA3.1-ST6GAL1 or empty vector and IκBα phosphorylation inhibitor (BAY 11–7085) or control.

**Additional file 3: Tables.**

## Data Availability

The data that support the findings of this study are available from the corresponding author upon reasonable request.

## Abbreviations

CCK8: Cell counting kit 8
CeRNA: Competing endogenous RNA
CI: Confidence interval
HCC: Hepatocellular carcinoma
HR: Hazard rate
lncRNA: Long non-coding RNA
NF-κB: Nuclear factor kappa B
OS: Overall survival
RFS: Recurrence-free survival
TINCR: Terminal differentiation-induced non-coding RNA
