# Supplementary figures and images for: Long noncoding RNA TINCR facilitates hepatocellular carcinoma progression and dampens chemosensitivity to oxaliplatin by regulating the miR-195-3p/ST6GAL1/NF-κB pathway
Source: J Exp Clin Cancer Res. 2022 Jan 3;41:5. doi: 10.1186/s13046-021-02197-x (PMC8722212; doi:10.1186/s13046-021-02197-x)

**A**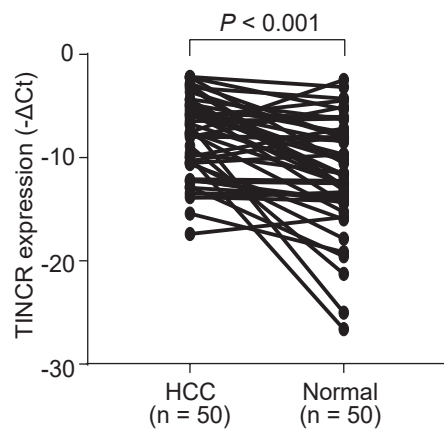**B**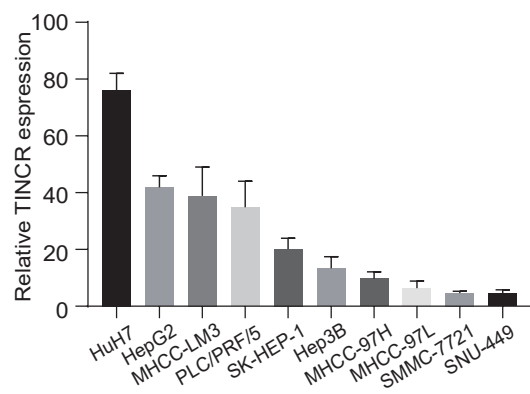**C**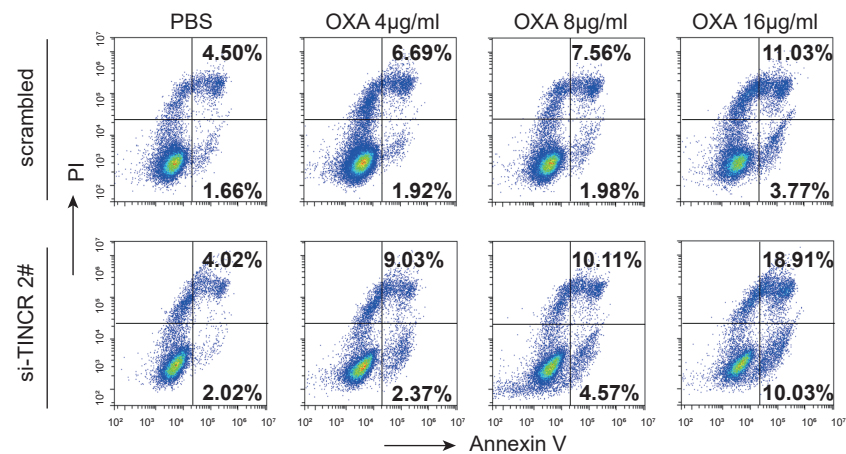**D**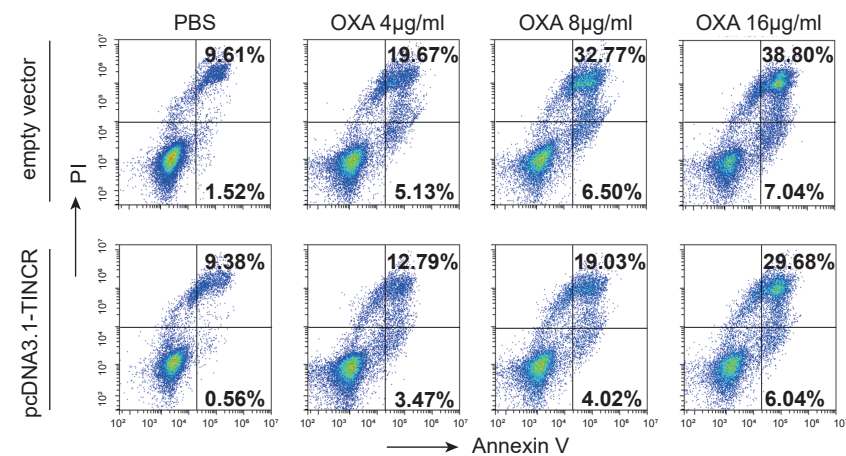

Supplement: Supplementary file 1 — Additional file 1: Figure S1 TINCR functions as an oncogenic lncRNA in HCC. A, Expression of TINCR in HCC compared to that in normal liver tissues (50 pairs). Paired t-test, P < 0.001. B, TINCR expression level in the 10 typical HCC cell lines. C-D, Representative images of apoptosis assay of increasing concentration of oxaliplatin in HuH7 cells transfected with si-TINCRs or scrambled control (C), and pcDNA3.1-TINCR or empty vector (D). [file 13046_2021_2197_MOESM1_ESM.pdf]

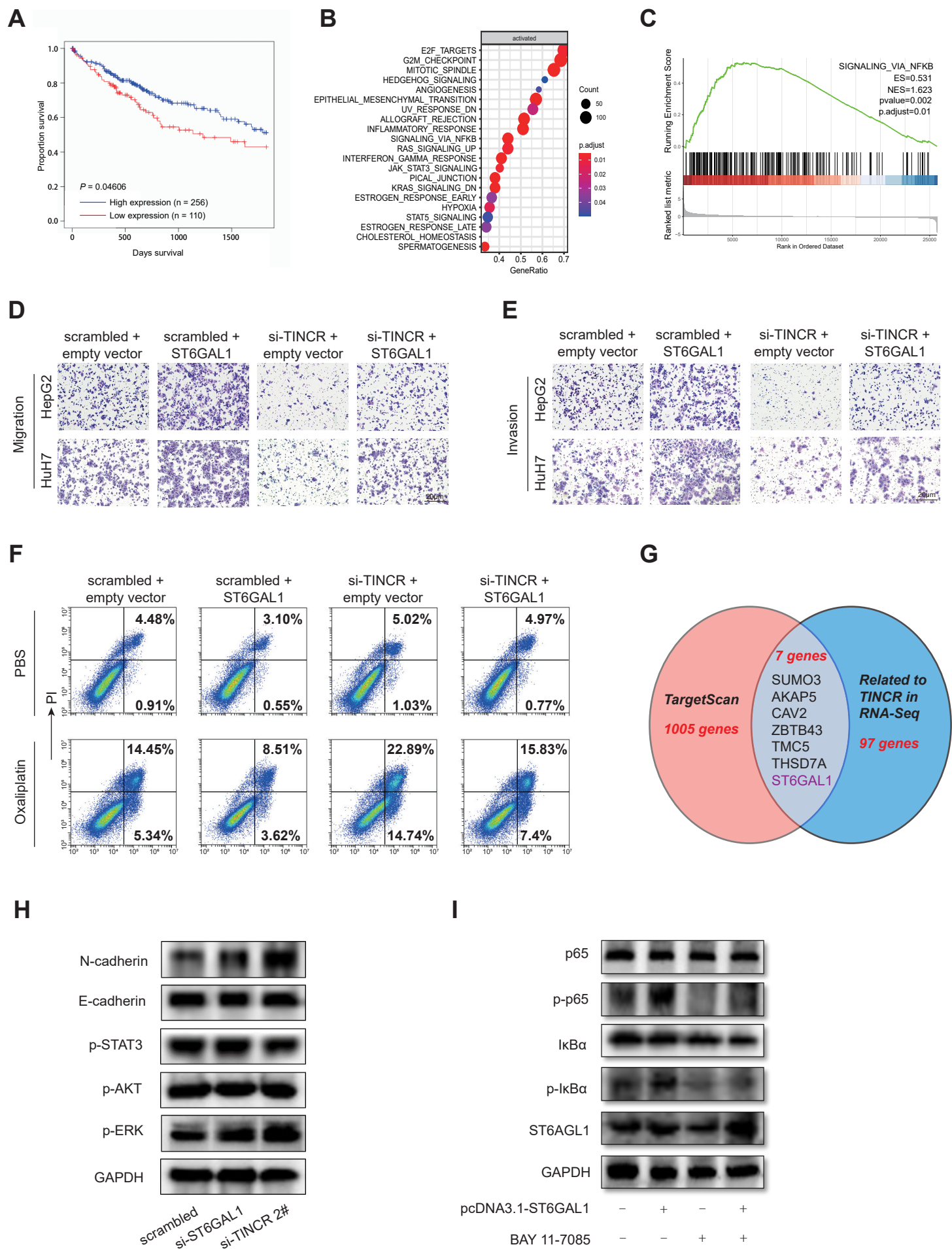

Supplement: Supplementary file 2 — Additional file 2: Figure S2. TINCR regulates HCC progression and oxaliplatin sensitivity through TINCR/miR-195-3p/ST6GAL1/NF kappa B Signaling. A, MiR-195-3p tends to be associated with good prognosis in the TCGA database. B-C, GSEA analysis based on TCGA data set shows a significant enrichment of high TINCR expression on gene sets related to several classical pathways (B), including NF kappa B pathway (C). D-E, Quantification of transwell migration (D) and invasion (E) assays in HepG2 and HuH7 cotransfected with si-TINCR 2# or scrambled control together with pc-DNA3.1-ST6GAL1 or empty vector. F, Representative images of the apoptosis assay of oxaliplatin (16 μg/ml) in the above-mentioned transfected HuH7 cells. G, 7 genes including ST6GAL1 are identified between the predicted target mRNAs in TargetScan and mRNAs that down-regulated with TINCR silencing in RNA-Seq. H, Western blot analysis of expression of key molecules in other typical pathways in HepG2 cells transfected with scrambled, si-ST6GAL1 or si-TINCR #2. I, Western blot analysis of expression of ST6GAL1 and NF-kappa-B-related markers in HepG2 cells cotransfected with pcDNA3.1-ST6GAL1 or empty vector and IκBα phosphorylation inhibitor (BAY 11–7085) or control. [file 13046_2021_2197_MOESM2_ESM.pdf]
