# Supplementary material for: Long noncoding RNA TINCR facilitates hepatocellular carcinoma progression and dampens chemosensitivity to oxaliplatin by regulating the miR-195-3p/ST6GAL1/NF-κB pathway
Source: J Exp Clin Cancer Res. 2022 Jan 3;41:5. doi: 10.1186/s13046-021-02197-x (PMC8722212; doi:10.1186/s13046-021-02197-x)
Supplement: Supplementary file 3 — Additional file 3: Tables. [file 13046_2021_2197_MOESM3_ESM.docx]

**Table S1.** Primer sequence used in RT-PCR.

| **Gene** | Sequence (5’ to 3’) |
| --- | --- |
| GAPDH | Forward - TGTGGGCATCAATGGATTTGG |
|  | Reverse - ACACCATGTATTCCGGGTCAAT |
| β-actin | Forward - AAGGAGCCCCACGAGAAAAAT |
|  | Reverse - ACCGAACTTGCATTGATTCCAG |
| U6 | Forward - CTCGCTTCGGCAGCACAU6 |
|  | Reverse - AACGCTTCACGAATTTGCGT |
| TINCR | Forward - TGTGGCCCAAACTCAGGGATACAT |
|  | Reverse - AGATGACAGTGGCTGGAGTTGTCA |
| ST6GAL1 | Forward - AACTCTCAGTTGGTTACCACAGA |
|  | Reverse - GGTGCAGCTTACGATAAGTCTT |

**Table S2.** Sequence used in siRNA and shRNA

|  | Sequence (5’ to 3’) |
| --- | --- |
| si-scramble | Forward - UUCUCCGAACGUGUCACGUTT |
|  | Reverse - ACGUGACACGUUCGGAGAATT |
| si-TINCR 1# | Forward - GCUGGCAUGUUCUGAAAUATT |
|  | Reverse - UAUUUCAGAACAUGCCAGCTT |
| si-TINCR 2# | Forward - GCUUCAAUACCUGCUACUUTT |
|  | Reverse - AAGUAGCAGGUAUUGAAGCTT |
| sh-scramble | Forward_CCGGTTCTCCGAACGTGTCACGTTTCAAGAGAACGTGACACGTTCGGAGAATTTTTTG |
|  | Reverse_AATTCAAAAAATTCTCCGAACGTGTCACGTTCTCTTGAAACGTGACACGTTCGGAGAA |
| sh-TINCR 1# | Forward_CCGGGCTGGCATGTTCTGAAATATTCAAGAGATATTTCAGAACATGCCAGCTTTTTTG |
|  | Reverse_AATTCAAAAAGCTGGCATGTTCTGAAATATCTCTTGAATATTTCAGAACATGCCAGC |
| sh-TINCR 2# | Forward_CCGGGCTTCAATACCTGCTACTTcTCAAGAGAAAGTAGCAGGTATTGAAGCTTTTTTG |
|  | Reverse_AATTCAAAAAAGCTTCAATACCTGCTACTTTCTCTTGAgAAGTAGCAGGTATTGAAGC |

**Table S3.** Baseline characteristics of the patients grouped by TINCR expression

| Variables | Low TINCR  N = 69 | High TINCR  N = 70 | *P* |
| --- | --- | --- | --- |
| Age, y |  |  | 0.673 |
| ≤ 50 | 35 (50.7) | 33 (47.1) |  |
| > 50 | 34 (49.3) | 37 (52.9) |  |
| Gender |  |  | 0.820 |
| Female | 8 (11.6) | 9 (12.9) |  |
| Male | 61 (88.4) | 61 (87.1) |  |
| Hepatitis B surface antigen |  |  | 0.480 |
| Negative | 8 (11.6) | 11 (15.7) |  |
| Positive | 61 (88.4) | 59 (84.3) |  |
| Liver cirrhosis |  |  | 0.351 |
| No | 37 (53.6) | 32 (45.7) |  |
| Yes | 32 (46.4) | 38 (54.3) |  |
| ALT, U/L |  |  | 0.046 |
| ≤ 40 | 46 (66.7) | 35 (50.0) |  |
| > 40 | 23 (33.3) | 35 (50.0) |  |
| AST, U/L |  |  | 0.105 |
| ≤ 40 | 42 (60.9) | 33 (47.1) |  |
| > 40 | 27 (39.1) | 37 (52.9) |  |
| ALBI grade |  |  | 0.021 |
| 1 | 43 (62.3) | 56 (80.0) |  |
| 2-3 | 26 (37.7) | 14 (20.0) |  |
| PLT, 10^9^/L |  |  | 0.118 |
| < 100 | 5 (7.2) | 11 (15.7) |  |
| ≥ 100 | 64 (92.8) | 59 (84.3) |  |
| AFP, ng/ml |  |  | 0.432 |
| ≤ 400 | 42 (60.9) | 38 (54.3) |  |
| > 400 | 27 (39.1) | 32 (20.0) |  |
| Largest tumor size, cm |  |  | 0.232 |
| ≤ 5 | 20 (29.0) | 27 (38.6) |  |
| > 5 | 49 (71.0) | 43 (61.4) |  |
| Tumor number |  |  | 0.511 |
| Single | 52 (75.4) | 56 (80.0) |  |
| Multiple | 17 (24.6) | 14 (20.0) |  |
| Macrovascular invasion |  |  | 0.592 |
| Absent | 63 (91.3) | 62 (88.6) |  |
| Present | 6 (8.7) | 8 (11.4) |  |
| Microvascular invasion |  |  | 0.188 |
| Absent | 42 (60.9) | 50 (71.4) |  |
| Present | 27 (39.1) | 20 (28.6) |  |
| Differentiation* |  |  | 0.441 |
| I - II | 30 (43.5) | 35 (50.0) |  |
| III - IV | 39 (56.5) | 35 (50.0) |  |

Data are presented as No (%).

*Tumour differentiation refers to criteria of Gleason classification.

Abbreviations: ALT, alanine aminotransferase; AST, aspartate aminotransferase; ALBI, albumin-bilirubin; PLT, platelet; AFP, alpha fetoprotein

**Table S4**. Univariate and multivariate analysis of risk factors for overall survival after curative liver resection in patients with hepatocellular carcinoma

| **Variables** | **Univariate** | | **Multivariate** | |
| --- | --- | --- | --- | --- |
|  | **HR (95% CI)** | ***P*** | **HR (95% CI)** | ***P*** |
| Age, y (> 50) | 0.75 (0.52-1.09) | 0.134 |  |  |
| Gender (male) | 1.96 (1.02-3.77) | 0.043 | 1.60 (0.82-3.12) | 0.172 |
| HBsAg (positive) | 1.82 (0.98-3.41) | 0.060 |  |  |
| Liver cirrhosis (yes) | 0.77 (0.53-1.13) | 0.181 |  |  |
| ALT, U/L (> 40) | 1.35 (0.92-1.97) | 0.121 |  |  |
| AST, U/L (> 40) | 1.53 (1.05-2.23) | 0.028 | 1.17 (0.76-1.79) | 0.483 |
| ALBI grade (2-3) | 1.63 (1.09-2.44) | 0.017 | 1.55 (1.02-2.34) | 0.039 |
| PLT, 109/L (< 100) | 0.54 (0.28-1.04) | 0.063 |  |  |
| AFP, ng/ml (> 400) | 1.00 (0.68-1.46) | 0.988 |  |  |
| Largest tumor size, cm (> 5) | 1.77 (1.17-2.68) | 0.007 | 1.60 (1.02-2.52) | 0.043 |
| Tumor number (multiple) | 1.79 (1.17-2.75) | 0.008 | 1.48 (0.94-2.32) | 0.089 |
| Macrovascular invasion (present) | 3.18 (1.77-5.73) | <0.001 | 2.66 (1.43-4.94) | 0.002 |
| Microvascular invasion (present) | 1.19 (0..80-1.77) | 0.380 |  |  |
| Differentiation (III - IV) | 1.56 (1.07-2.28) | 0.022 | 1.30 (0.86-1.98) | 0.213 |
| TINCR expression (High) | 1.85 (1.26-2.70) | 0.002 | 2.00 (1.35-2.94) | <0.001 |

Abbreviations: HBsAg, hepatitis B surface antigen; ALT, alanine aminotransferase; AST, aspartate aminotransferase; ALBI, albumin-bilirubin; PLT, platelet; AFP, alpha fetoprotein; HR, hazard rate; CI, confidence interval

**Table S5**. Univariate and multivariate analysis of risk factors for recurrence-free survival after curative liver resection in patients with hepatocellular carcinoma

| **Variables** | **Univariaate** | | **Multivariate** | |
| --- | --- | --- | --- | --- |
|  | **HR (95% CI)** | ***P*** | **HR (95% CI)** | ***P*** |
| Age, y (> 50) | 0.74 (0.52-1.07) | 0.108 |  |  |
| Gender (male) | 1.52 (0.87-2.67) | 0.141 |  |  |
| HBsAg (positive) | 1.71 (0.96-3.05) | 0.070 |  |  |
| Liver cirrhosis (yes) | 0.85 (0.59-1.22) | 0.383 |  |  |
| ALT, U/L (> 40) | 1.53 (1.06-2.22) | 0.022 | 1.12 (0.75-1.89) | 0.464 |
| AST, U/L (> 40) | 1.82 (1.26-2.63) | 0.001 | 1.46 (0.92-2.34) | 0.112 |
| ALBI grade (2-3) | 1.71 (1.16-2.54) | 0.007 | 1.92 (1.27-2.91) | 0.002 |
| PLT, 109/L (< 100) | 0.59 (0.32-1.11) | 0.101 |  |  |
| AFP, ng/ml (> 400) | 0.99 (0.68-1.43) | 0.951 |  |  |
| Largest tumor size, cm (> 5) | 1.89 (1.27-2.81) | 0.002 | 1.80 (1.17-2.78) | 0.008 |
| Tumor number (multiple) | 1.61 (1.05-2.45) | 0.028 | 1.22 (0.78-1.91) | 0.375 |
| Macrovascular invasion (present) | 3.16 (1.78-5.60) | <0.001 | 3.16 (1.71-5.85) | <0.001 |
| Microvascular invasion (present) | 1.36 (0.92-1.99) | 0.120 |  |  |
| Differentiation (III - IV) | 1.37 (0.95-1.98) | 0.092 |  |  |
| TINCR expression (High) | 1.51 (1.05-2.18) | 0.027 | 1.60 (1.09-2.35) | 0.016 |

Abbreviations: HBsAg, hepatitis B surface antigen; ALT, alanine aminotransferase; AST, aspartate aminotransferase; ALBI, albumin-bilirubin; PLT, platelet; AFP, alpha fetoprotein; HR, hazard rate; CI. confidence interval

**Table S6**. Prognostic model scoring system

| **Variables** | **Score** |
| --- | --- |
| ALBI grade |  |
| 1 | 0 |
| 2-3 | 1 |
| Largest tumor size, cm |  |
| 5 | 0 |
| > 5 | 1 |
| Macrovascular invasion |  |
| Absent | 0 |
| Present | 1 |
| TINCR expression |  |
| Low | 0 |
| High | 1 |
| **Risk stratification** |  |
| Low risk | 0-1 |
| Intermediated risk | 2-3 |
| High risk | 4-5 |

Abbreviations: ALBI, album-bilirubin
